# Controllable quantum point junction on the surface of an antiferromagnetic topological insulator

**DOI:** 10.1038/s41467-021-24276-5

**Published:** 2021-06-28

**Authors:** Nicodemos Varnava, Justin H. Wilson, J. H. Pixley, David Vanderbilt

**Affiliations:** 1 Department of Physics & Astronomy, Center for Materials Theory, Rutgers University, Piscataway, NJ USA; 2 Center for Computational Quantum Physics, Flatiron Institute, New York, NY USA; 3 Physics Department, Princeton University, Princeton, NJ USA

**Keywords:** Electronic properties and materials, Magnetic properties and materials, Topological defects, Topological insulators

## Abstract

Engineering and manipulation of unidirectional channels has been achieved in quantum Hall systems, leading to the construction of electron interferometers and proposals for low-power electronics and quantum information science applications. However, to fully control the mixing and interference of edge-state wave functions, one needs stable and tunable junctions. Encouraged by recent material candidates, here we propose to achieve this using an antiferromagnetic topological insulator that supports two distinct types of gapless unidirectional channels, one from antiferromagnetic domain walls and the other from single-height steps. Their distinct geometric nature allows them to intersect robustly to form quantum point junctions, which then enables their control by magnetic and electrostatic local probes. We show how the existence of stable and tunable junctions, the intrinsic magnetism and the potential for higher-temperature performance make antiferromagnetic topological insulators a promising platform for electron quantum optics and microelectronic applications.

## Abstract (web summary)

Recent advances in the identification and growth of antiferromagnetic topological insulators open the way to the manipulation of the chiral edge states that are topologically required at their step edges and domain walls. Here, the authors propose a quantum point junction formed by two types of edge states and discuss its applications in electron quantum optics.

## Introduction

Surface and edge-state engineering of topological materials offers great promise for future electronic devices. Owing to the topological properties of the bulk of the material, surface states emerge that are protected from elastic and inelastic scattering. In particular, the community realized early on that topologically-protected chiral (one-way) or helical (two-way) edge states provide dissipationless “quantum wires”^1,2^ with potential applications in sensor, low-power electronics, and quantum information technologies. A crucial part of engineering such wires requires robust and tunable junctions between edge states.

Strikingly, chiral edge states provide directional control of carrier propagation and (topological) protection against impurity backscattering. This was first demonstrated for quantum Hall edge states in 2D electron gases, but these systems require very low temperature and external magnetic fields. A potentially more practical approach to engineering chiral edge states is at the boundary of a 2D quantum anomalous Hall or “Chern” insulator^3^. Experimentally this was first realized in thin films of magnetically doped topological insulators (TI)^4^. Unfortunately the inhomogeneity of the magnetic dopants leads to inevitable disorder^5^ and as a result the quantized response is observed at much lower temperatures than the magnetic gap and Curie temperature allow; to date the state of the art is around ~1*K*^6–9^. More recently, the discovery of the quantum anomalous Hall effect in twisted bilayer graphene^10,11^, MnBi_2_Te_4_^12^ and MnBi_2_Te_4_/Bi_2_Te_3_ heterostructures^13^ holds promise for the realization of topologically protected chiral channels at higher temperatures, due in part to the absence of magnetic-impurity disorder^14^.

In their bulk version, the MnBi_2_Te_4_ family of materials belongs to a class of 3D materials that have been variously described as intrinsic magnetic topological insulators^12,15–17^, axion insulators^18–20^ and second-order topological insulators^21–23^. The essential idea is to identify a material whose magnetic symmetry group enforces^20^ a quantized bulk axion coupling^24,25^ of *θ* = *π*, as in an ordinary 3D TI, but does not enforce the presence of gapless surface states. Instead, gapped surfaces can appear naturally on such materials. When they do, they exhibit a half-quantized surface anomalous Hall conductivity, i.e., an odd integer times *e*^2^/2*h*, whose sign is determined by details of the magnetic order at the terminating surface. Thus, manipulation of the surface termination and/or magnetic order in one region of the surface relative to a neighboring patch, or on one facet relative to another that meets it at a “hinge,” can give rise to a chiral edge channel at the boundary between these patches or facets^19^.

In this work, we develop a theoretical prescription for the creation and manipulation of chiral edge channels on the surface of an antiferromagnetic (AFM) TI. This class of materials was introduced theoretically by Mong and Moore^26^ and has recently become the focus of intense research with various candidates such as MnBi_2_Te_4_^27^, MnBi_4_Te_7_^28^, EuIn_2_As_2_^29^ and NpBi^30^ appearing in the literature. Motivated by these recent developments and the fact that there is in principle no reason why both the bulk and surface gaps could not be on the order of hundreds of meV, allowing for potential high temperature device operation for certain applications, we propose and explore the properties of a robust and controllable quantum point junction (QPJ) on the surface of an AFM TI.

**Fig. 1:**
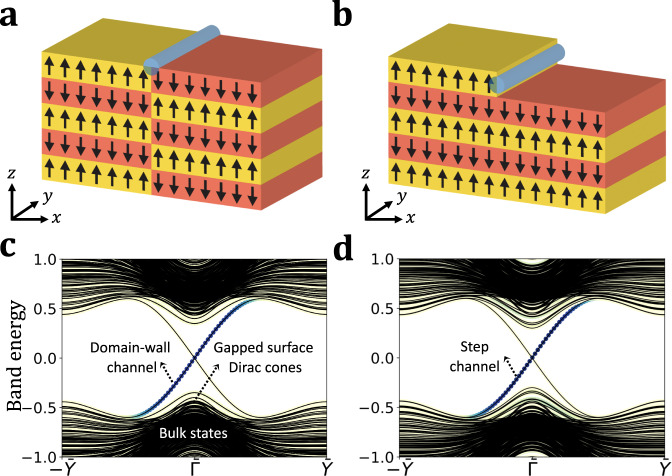
Types of chiral channels at surface of an A-type antiferromagnetic topological insulator (AFM TI). Depiction of the chiral channel (blue cylinder) due to (**a**), a bulk domain wall (**b**), a surface step. Surface band structures along (001) in the presence of (**c**), a bulk domain wall (**d**), a surface step. The projection of the states on the chiral channels (blue cylinder) in (**a**), (**b**), are also shown (blue markers) to illustrate the localization of the massless Dirac fermions that disperse linearly along the channel direction at low energy with velocities (**c**), *v*_dw_ and (**d**), *v*_st_. The description of the model Hamiltonian can be found in “Methods”. Energies are expressed in terms of the onsite energy *m* in Eq. (8).

Figure 1a, b shows a prototypical spin arrangement in an AFM TI. The magnetic ordering is A-type AFM, i.e., with magnetization uniform in-plane but alternating from plane to plane along the stacking direction, which we take to be along $\hat{{\bf{z}}}$. As described in Ref. ^26^, each individual layer can be thought of as adiabatically connected to a 2D Chern insulator, with the sign of the Chern number alternating from layer to layer. The sign of the surface anomalous Hall conductivity of ±*e*^2^/2*h* is thus determined by the magnetic orientation of the last layer at the surface. As a result, two kinds of 1D chiral channels can occur at the surface. As shown in Fig. 1a, the emergence of a bulk AFM domain wall at the surface reverses the sign of the anomalous Hall conductivity on either side of the resulting line defect, which therefore carries a topologically protected chiral channel we refer to as a domain-wall channel. Alternatively, even if no bulk AFM domain walls are present, a single-height step can occur on the surface, as shown in Fig. 1b. If it does, it also marks a sign reversal of the anomalous Hall conductivity when crossing the step, and thus carries a chiral edge channel as well. We will refer to this as a step channel.

Figure 1c, d shows the manifestation of the domain-wall and step channel in the surface band structure as described in the context of a tight-binding model used throughout this work (see “Methods”). The presence of either of these defects results in 1D linear dispersions in the otherwise gapped bulk and surface spectrum of the AFM TI. The states that comprise the chiral bands are exponentially localized in the vicinity of the channel, and host 1D massless Dirac fermions.

**Fig. 2:**
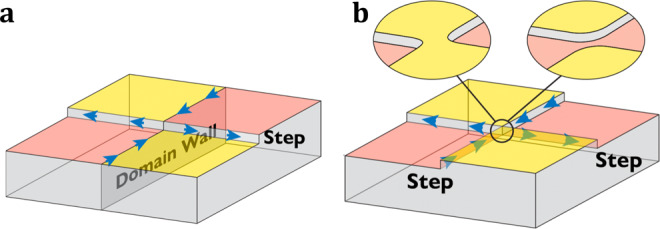
Stable and unstable junctions. **a** The intersection of a domain-wall channel with a step channel results in a thermodynamically stable junction, i.e., small surface deformations can only move the junction but not remove it. **b** The intersection of two step channels (or two domain-wall channels) is unstable. The inset shows how small deformations remove the junction. Blue arrows indicate the direction of propagation on the chiral channels, while orange and yellow surfaces indicate whether the anomalous Hall conductivity is ± *e*^2^/2*h* respectively.

The opportunity opened by the presence of two different kinds of 1D chiral channels at the surface is that these can be made to intersect, as shown in Fig. 2a, and such intersections are expected to remain thermodynamically stable. In contrast, as illustrated in Fig. 2b, an intersection between two surface steps can easily evolve via a pinch-off event into a configuration in which an isthmus of constant surface height separates the steps; indeed, the width of such an isthmus will tend to grow due to the line tensions of the steps, and the quantum junction will be removed. A similar mechanism affects the intersection of two domain walls^31^. In fact, setups like those depicted in the inset of Fig. 2b, where two chiral channels come in close proximity, have long been used in quantum Hall systems to realize electron interferometers^32,33^. These constructions, known as quantum point contacts (QPCs)^34,35^, enable tunneling between channels, and were recently used to observe the braiding of anyons^36^.

Our proposal aims to highlight a robust way to construct intersecting chiral channels by making use of a material system that is on the verge of discovery. In fact, these junctions were recently observed to appear naturally at the surface of the putative AFM TI MnBi_2_Te_4_^37^. Moreover, we show that a QPJ can be controlled by scanning tips of the kind used in scanning tunneling microscopy (STM) and related methods. Here, we are interested in local probes that affect the magnetic moments and electrostatic potential, which we refer to as magnetic and electrostatic STM tips respectively. We explore the properties of the QPJ by constructing the Hamiltonian associated with the system depicted in Fig. 2a and performing dynamic wave-packet (WP) simulations that allow us to extract the *S*-matrix of the junction. Remarkably, we find that magnetic and electrostatic STM tips in proximity with the junction can, in principle, be used to realize any unitary *S*-matrix. In addition, we show that the effect of symmetry breaking terms and weak disorder can be “gauged away” using the two tips.

The stability and tunability of the proposed junction, together with the intrinsic benefits of a magnetic topological material, can be utilized in applications involving unidirectional channels, such as electron interferometry, low-power electronics and quantum information processing.

## Results

### Extracting the *S* matrix

**Fig. 3:**
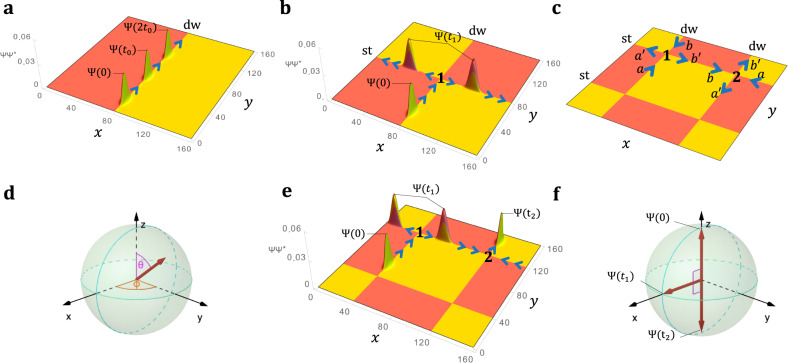
Effective description of wave packet (WP) time evolution. **a** Snapshots of the WP showing the propagation on the domain-wall channel. **b** A WP scatters at Junction 1 and splits into two spatially separated outgoing components of the wavefunction. **c** Channel labeling convention for Junction 1 and 2. **d** Qubit representation of a WP state on the Bloch sphere. **e** The initial WP splits after encountering Junction 1, the two components then meet at Junction 2, interfering destructively on channel $a^{\prime}$ and constructively on channel $b^{\prime}$. **f** Qubit representation of the time evolution in (**e**). WP plots in (**a**), (**b**), (**e**), are calculated from the (001)-projected probability densities at different times. Time-evolution animations of the WPs in (**a**), (**e**) are shown Supplementary Movies 1, 2.

We begin by considering the WP dynamics at the surface of an AFM TI. Figure 3a shows the calculated time evolution of a WP on a single domain-wall channel, while Fig. 3b, that of a WP in the presence of the QPJ in Fig. 2a (see also Supplementary Movies for time-evolution animations). In both cases the dissipationless channels are protected from back-scattering by the insulating bulk and surface gaps. The wave function of the WP is thus exponentially confined to the vicinity of the one-dimensional channel, and it travels with a constant group velocity along the channel. In Fig. 3b, a WP enters along the domain-wall channel, gets split by the QPJ, and then the two components travel away from the QPJ along the step channels. Later we shall consider configurations in which multiple consecutive scattering events occur.

To understand the behaviors observed above, we note that the wave function of a WP propagating along a single domain-wall channel in direction *y*, as in Fig. 3a, can be well approximated, see Supplementary Methods, as

1 $${{{\Psi }}}_{\sigma \tau }^{{\rm{dw}}}(x,y,z,t)={\chi }_{\sigma \tau }^{{\rm{dw}}}(x,z)f(y-{y}_{0}-{v}_{{\rm{dw}}}t)\ .$$

Here ${\chi }_{\sigma \tau }^{{\rm{dw}}}(x,z)$ captures the transverse shape (*x*, *z*) and spin-orbital character (*σ*, *τ* indices respectively) of the WP, see Supplementary Methods, while *f*(*y*) is the envelope function of the WP, which we take to be a Gaussian. The WP is launched from position *y*_0_ at time *t* = 0 and travels with group velocity *v*_dw_ (which is set by the surface state dispersion in Fig. 1c). In modeling at this level we neglect spreading of the WP, which we find to be negligible in our simulations. Similar considerations apply to the propagation of a WP on a step along *x* traveling with group velocity *v*_st_ (that is set by the surface state dispersion in Fig. 1d).

We now consider the scattering event depicted in Fig. 3b, where an incoming WP splits after encountering the QPJ. We will use unprimed labels *a* and *b* to refer to the two incoming domain-wall channels of Junction 1, as in Fig. 3c. Note that the extra junctions are the result of in-plane periodic boundary conditions. The incoming initial conditions are specified by amplitudes *ϕ*_*a*_ = 1 and *ϕ*_*b*_ = 0. Now let *t*_1_ indicate a time after the scattering through Junction 1 is complete, but before Junction 2 is encountered. We label the two outgoing step channels as $a^{\prime}$ and $b^{\prime}$, adopting once and for all the arbitrary convention that $a\to a^{\prime}$ and $b\to b^{\prime}$ result from taking left turns, as shown in Fig. 3c. As illustrated in Fig. 3b, one component of the WP moves to the right and the other to the left, with velocities *v*_st_ and −*v*_st_ respectively. At time *t*_1_ both will be centered at a distance *x*_1_ relative to the junction, so in general we expect to find

2 $${{{\Psi }}}_{\sigma \tau }^{{\rm{st}}}(x,y,z,{t}_{1})=	\; {\phi }_{a^{\prime} }{\tilde{\chi }}_{\sigma \tau }^{{\rm{st}}}(y,z)f(x+{x}_{1})\\ 	 +{\phi }_{b^{\prime} }{\chi }_{\sigma \tau }^{{\rm{st}}}(y,z)f(x-{x}_{1})\ .$$

Here ${\phi }_{a^{\prime} }$ and ${\phi }_{b^{\prime} }$ are the amplitudes (magnitude and phase) describing scattering from incoming channel *a* into channels $a^{\prime}$ and $b^{\prime}$ respectively, and ${\tilde{\chi }}^{{\rm{st}}}$ is the time-reversed partner of *χ*^st^. These expectations are well reproduced in our full numerical calculations which therefore allow us to extract the amplitudes ${\phi }_{a^{\prime} }$ and ${\phi }_{b^{\prime} }$.

Similar calculations, where the incident WP approaches Junction 1 along the $-\hat{y}$ direction on channel *b*, allow us to extract the corresponding amplitudes that result for initial conditions of *ϕ*_*a*_ = 0 and *ϕ*_*b*_ = 1. Thus, we can model a combined scattering event via

3 $$\left(\begin{array}{l}{\phi }_{a^{\prime} }\\ {\phi }_{b^{\prime} }\\ \end{array}\right)=S\left(\begin{array}{l}{\phi }_{a}\\ {\phi }_{b}\\ \end{array}\right)$$

where the elements of the S-matrix are determined by the four complex amplitudes discussed above.

In this way, the evolution of the system of propagating WPs is mapped onto that of a two-level quantum system, so that it is enough to restrict *S* to be an SU(2) matrix. The characterization of a junction by such an S-matrix is a central element of our theory. It is illustrative to represent the initial or final state as a point on the Bloch sphere,

4 $$\left(\begin{array}{l}{\phi }_{a}\\ {\phi }_{b}\\ \end{array}\right)=\left(\begin{array}{l}\cos (\theta /2)\\ {e}^{i\phi }\sin (\theta /2)\\ \end{array}\right),$$

where *θ* determines the relative WP magnitude on channels *a* and *b* and *ϕ* their phase difference, as illustrated in Fig. 3d.

Each junction scattering event can then be described by the action of the corresponding junction S-matrix on the spinor representation of the channel states, regarded as a qubit state, and the result of consecutive QPJ scattering events, as in Fig. 3e, corresponds to the action of consecutive gates acting on these qubits as illustrated in Fig. 3f.

Let us now return to a more specific discussion of our full time-evolution calculations, and our analysis of them in terms of the framework sketched above. Figure 3e shows the time evolution of a WP initiated on channel *a*. The WP propagates toward and then scatters at Junction 1, splitting into two equal parts. Later the two WPs pass through Junction 2, interfering destructively and constructively on outgoing channels $a^{\prime}$ and $b^{\prime}$ respectively. As promised, we can describe the time evolution of the WP configuration as a qubit passing through two gates. Indeed, using the convention of Fig. 3c, the calculated *S* matrix of Junction 1 and 2 corresponds to the Hadamard gate

5 $${S}_{1}={S}_{2}=\frac{1}{\sqrt{2}}\left(\begin{array}{ll}1&-1\\ 1&1\\ \end{array}\right),$$

so that the final state is related to the initial one by applying the Hadamard gate twice. Geometrically the *S* matrix expressed as

6 $$S={R}_{\hat{{\bf{n}}}}(\varphi )={e}^{-i\frac{\varphi }{2}\hat{{\bf{n}}}\cdot {\boldsymbol{\sigma }}}$$

describes a qubit rotation by an angle *φ* through an axis $\hat{{\bf{n}}}$ and ***σ*** = (*σ*_*x*_, *σ*_*y*_, *σ*_*z*_) is a vector of Pauli matrices. Since ${S}_{1}={S}_{2}={R}_{\hat{{\bf{y}}}}(\pi /2)$, each application rotates the qubit by 90^∘^ around the $\hat{{\bf{y}}}$ axis of the Bloch sphere, resulting in an overall reversal of the pseudospin as shown in Fig. 3f.

### Controlling the *S* matrix

Before explaining how the control of the *S* matrix is achieved, it is illustrative to break down the action of *S* into three stages. First we have the propagation along the incoming channels; since these cannot scatter into one another, this is represented by a diagonal matrix *S*_dw_. Then there is the scattering *S*_pj_ at the QPJ itself, followed by another channel-diagonal propagation *S*_st_ on the outgoing step channels. The overall *S* matrix can then be written in terms of the Pauli matrices as

7 $$S={S}_{\text{st}}{S}_{\text{pj}}{S}_{\text{dw}}={e}^{-i\gamma {\sigma }_{z}/2}{e}^{-i\beta {\sigma }_{y}/2}{e}^{-i\alpha {\sigma }_{z}/2},$$

where *S*_pj_ is expressed as a real orthogonal matrix because the phases can be absorbed into *S*_dw_ and *S*_st_. Remarkably, (*α*, *β*, *γ*) are exactly the three Euler angles that can be used to express any SU(2) matrix. Thus control over the three Euler angles results in a universally programmable gate, which we now demonstrate.

To control the *S* matrix, we will use two local probes in the vicinity of the junction. The first one, which we refer to as a magnetic STM tip, affects the local magnetic moments, and as we shall see, controls the magnitudes of the *S* matrix. The second probe is an electrostatic STM tip modifying the site energies under the tip, thus controlling the phases of the *S* matrix. The effect of the magnetic tip is controlled through the coefficient *V*_Z_, and that of the electrostatic tip through *V*_G_, both acting in the local vicinity of the junction. For more details on how this is modeled, see Eq. (9) in “Methods”.

### Magnitude control

**Fig. 4:**
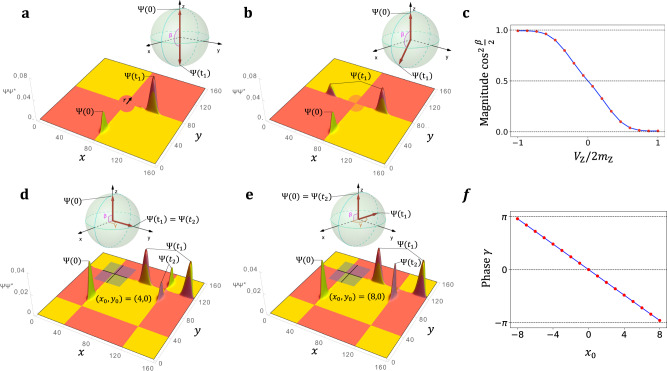
Magnitude and phase manipulation of the quantum point junction. **a** The magnetic tip with *V*_Z_ = 2*m*_Z_ in Eq. (9) (see “Methods”), has polarized the surface spins in a circular region centered at the junction resulting in two uncoupled channels. **b** Partially polarized region with *V*_Z_ = 0.4*m*_Z_ causes unequal splitting of the wave packet (WP). **c** Numerical calculation of the magnitude splitting ${\cos }^{2}\beta /2$ as a function of *V*_Z_. Each value corresponds to the integral of ∣Ψ(*t*_1_)∣^2^ on channel $a^{\prime}$ of Junction 1. **d**, **e** Applying the electrostatic tip with *V*_G_ = 0.6 in Eq. (9), at Junction 1 induces a phase difference between the outgoing WPs which then affects how they interfere at Junction 2. **d** The center of the rectangular region Ω_G_ is chosen so that *γ* = *π*/2. **e**, Same but we set *γ* = *π* making the WPs constructively (destructively) interfere on channel *a*(*b*) of Junction 2. **f** Numerical calculation of the angle *γ* from the relative phase of the outgoing WPs at *t*_1_ as a function of *x*_0_. The phases of the outgoing WPs are determined from the inner product between Ψ(*t*_1_) in the presence and absence of the phase gate. Time-evolution animations of the scattering events in (**a**), (**b**), (**d**), (**e**), are shown in Supplementary Movies 3,4,5,6.

We set *V*_G_ = 0, leaving the electric potential constant throughout the crystal so that no extra phase evolution occurs during the propagation (*α* = *γ* = 0), and we vary the strength of the magnetic tip *V*_Z_. This affects the left-right magnitude splitting, i.e., $S={R}_{\hat{{\bf{y}}}}(\beta )={e}^{-i\beta {\sigma }_{y}/2}$ in Eq. (7), with *β* = *β*(*V*_Z_). To understand the mechanism behind the magnitude control, first consider the extreme scenario depicted in Fig. 4a. Here a strong magnetic STM tip has polarized the surface magnetization in the vicinity of the junction (orange circular region), forcing the anomalous Hall conductivity to be uniformly + *e*^2^/2*h* in that area (see “Methods”). This essentially “removes” the junction, and the WP is completely transferred from the domain-wall (channel *a*) to the edge of the step (channel $b^{\prime}$), so that ${S}_{1}={R}_{\hat{{\bf{y}}}}(\pi )$. An example of partial polarization, is shown in Fig. 4b, while the results of tuning *V*_*Z*_ over the entire range of tip strength is shown in Fig. 4c, where we plot the numerically calculated value of ${\cos }^{2}(\beta /2)$, which represents the asymmetry between left- and right-scattered WPs, as a function of *V*_Z_. This demonstrates the universal control of the Euler angle *β* using a magnetic STM tip. In the Supplementary Discussion we consider a strong magnetic STM tip that decouples the channels inducing a QPC and we vary the area of the region applied to analyze tunneling between the channels.

### Phase control

To illustrate the phase control, we set *V*_Z_ = 0, fix *V*_G_ to a non-zero value (see Eq. (9) in “Methods”), and control the position of the electrostatic tip. Then ${S}_{j}={R}_{\hat{{\bf{z}}}}(\gamma ){R}_{\hat{{\bf{y}}}}(\pi /2){R}_{\hat{{\bf{z}}}}(\alpha )$, where *α* and *γ* are determined by the position (*x*_0_, *y*_0_) of the tip relative to the junction, as described by Eq. (10) (see “Methods”). The electrostatic tip is depicted as a shaded square with origin (*x*_0_, *y*_0_) in Fig. 4d, e. In fact, our choice of *ϕ*_*a*_ = 1 and *ϕ*_*b*_ = 0 simplifies the situation, since ${R}_{\hat{{\bf{z}}}}(\alpha )$ just corresponds to an overall phase, which is not of interest. Physically, the WP splits equally at the first junction (*β* = *π*/2), and the electrostatic STM tip, corresponding to the second term in Eq. (9), is then used to control the relative phases of the outgoing WPs via the ${R}_{\hat{{\bf{z}}}}(\gamma )$ term.

In Fig. 4d–f, we illustrate the phase control by applying the electrostatic gate on Junction 1. To see the effects of the phase manipulation, we consider the interference that conveniently occurs when the WPs meet again (due to periodic boundary conditions in *x* and *y*) at Junction 2. In Fig. 4d, the electrostatic tip is centered four unit cells to the right at (*x*_0_, *y*_0_) = (4, 0), which approximately makes *γ* = *π*/2 so that ${S}_{1}={R}_{\hat{{\bf{z}}}}(\pi /2){R}_{\hat{{\bf{y}}}}(\pi /2)$, while ${S}_{2}={R}_{\hat{{\bf{y}}}}(\pi /2)$ as before. After scattering at Junction 1 the outgoing WP, whose state corresponds to a vector pointing along the +*y* direction of the Bloch sphere, becomes the incoming WP at Junction 2. Since Junction 2 acts as a rotation around the *y*-axis, it does not affect the qubit state of the WP. Similarly, in Fig. 4e, we set (*x*_0_, *y*_0_) = (8, 0), so that after encountering Junction 1 the qubit state points along $-\hat{{\bf{x}}}$, and after Junction 2 it returns to its initial $+\hat{{\bf{z}}}$ state. In Fig. 4f, we present a numerical calculation of *γ* versus *x*_0_. This is done by calculating the phase of the WPs just after it scatters off Junction 1. We find a linear behavior as expected from Eq. (10).

In summary, using the two STM tips we can control *α*, *β*, and *γ* independently in Eq. (7), so that the junction can be made to implement any SU(2) gate.

### Symmetries

Our QPJ has some features that even though do not effect our main conclusions, they should nevertheless not be expected in any real application. For example, the QPJ naturally implements the Hadamard gate and the group velocities of the domain-wall and step channel are approximately equal Fig. 1c, d. In the former case, this behavior is enforced by the geometry of the junction and the mirror symmetries *M*_*x*_ and *M*_*y*_ of the bulk Hamiltonian. In the latter case, the velocity anisotropy is small due to the simplicity of the model. In the Supplementary Discussion we introduce mirror and particle-hole symmetry breaking terms to remove these non-essential features and show that they do not affect the ability of our protocol to define and control the *S* matrix.

### Stability to disorder

A significant advantage of the QPJ design presented here is that the chiral channels on the domain walls and steps cannot back-scatter and are therefore expected to be robust against the presence of weak disorder (i.e., such that the average bulk and surface gaps remains open). We demonstrate this topological protection of the QPJ by introducing disorder into our model via a short-ranged random potential that is sampled from a Gaussian distribution. Although the qubit gets dephased in a different way for each realization of disorder, as we demonstrate in the Supplementary Discussion, the electrostatic tip can be used to recalibrate the QPJ. This allows us to remove the random offsets arising from the specific impurity configuration, thus enabling the control of the junction even in the presence of weak disorder.

## Discussion

**Fig. 5:**
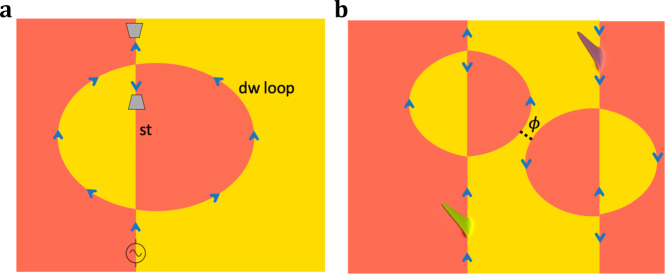
Illustrations of device implementations at the surface of an antiferromagnetic topological insulator. **a** A Mach-Zehnder electron interferometer consisting of a domain-wall channel loop intersected by a step. Arrows show the direction of propagation while trapezoids represent detectors. **b** Two-qubit entanglement can be achieved by coupling two of the interferometers through the Coulomb interaction to realize a controlled phase-shift gate.

In this work, we propose a versatile platform for performing electron quantum optics^38,39^. It is not hard to see how existing constructions, such as the Mach-Zehnder electron interferometer^33^, can be implemented directly on the surface of an AFM TI. Figure 5a shows a domain-wall loop channel intersecting a step channel. In this case, the interferometer works by splitting the incoming current (that flows on the step channel) in two parts that encircle the area defined by the domain-wall loop and meet at the second QPJ where they interfere. An Aharonov-Bohm phase can be introduced by threading the loop with a magnetic flux. In this way, varying the external magnetic field results in oscillations of the output conductance. The ability to control the *S* matrix of the QPJs means they can be calibrated so that the interferometer can be used as a sensitive sensor.

In the quantum-Hall regime of 2D electron gases, the long edge-state coherence length and the on-demand creation of indistinguishable, single-electron WPs^40–44^ have inspired ambitious proposals that consider electron interferometers as platforms for quantum information processing^45–47^. In this approach, electronic flying qubits^48^ – another prominent scheme is based on photonic flying qubits^49^—are subjected to quantum operations while they are being coherently transferred, providing control over qubit separation and non-local entanglement. In contrast to photons, electrons are subject to Coulomb interactions, making them vulnerable to dephasing but, at the same time, allowing control of the entanglement strength and manipulation of the phase^50^.

In the context of quantum Hall systems, entangling devices have been constructed^51^ by Coulomb-coupling two Mach-Zehnder interferometers to induce a relative phase *ϕ* between the WPs of the two coupled channels. These devices can be used as electronic quantum erasers^51,52^ or even as entangling quantum gates, i.e., controlled phase gates^46^. In fact, since we have shown that QPJs can implement any single-qubit gate, a gate such as that in Fig. 5b, can be adopted to perform the two-qubit entanglement for a universal set of quantum gates. We also remark that chiral Majorana fermions, first seen in magnetic TI-superconductor structures^53^ and more recently in topological superconductors^54–56^, are the superconducting analog of the chiral fermions discussed here. It has been proposed that topological quantum computing can be achieved using WPs propagating on chiral Majorana channels^57^. An interesting question is whether analogs of robust QPJs can be constructed in the superconducting case.

Finally, we comment on issues of temperature of operation and decoherence. In one sense, our scheme is robust to higher temperatures than quantum Hall systems; we only require the operating temperature to be small compared to the Neel temperature and the band gap in which the chiral mode is propagating. For example, surface Dirac gaps of up to 100 meV have recently been observed in ARPES measurements of Sb-doped MnBi_2_Te_4_^58^. While these considerations indicate that operation at tens of Kelvins should be possible, we are keenly aware that any application that is sensitive to decoherence will require lower temperatures, perhaps comparable to those needed for the quantum Hall platform, to avoid dephasing due to electron-electron, electron-phonon, and electron-magnon interactions.

In the quantum Hall context, the coherence length *l*_*ϕ*_ was determined by tracking the appearance of quantum interference in Mach-Zehnder interferometers^59,60^. These experiments showed that *l*_*ϕ*_ scales inversely with temperature, with the dominant dephasing mechanism attributed to electron-electron interactions between adjacent edge channels while short-range and long-range Coulomb interactions within each channel are sub-leading^61^. At low temperatures, the coherence length was even found to reach macroscopic scales (*l*_*ϕ*_ = 250 μm)^62^. Our work points to the need to characterize chiral edge channels on AFM TIs in a similar way. An implementation of the Mach-Zehnder interferometer, as in Fig. 5a, would provide a means of quantifying the dephasing mechanisms in magnetic topological materials, potentially opening a path to utilizing their unique properties in future quantum information science applications.

## Methods

### Model Hamiltonian

We consider an adaptation of a simple four-band tight-binding model proposed by Bernevig et al.^63,64^ to describe systems exhibiting a topological phase transition mediated by a single band inversion at Γ. The simplicity of the model makes detailed calculations practical even for large systems. The model is written in terms of two spinful orbitals per lattice site and takes the form

8 $${H}_{0}=	\; m\mathop{\sum}\limits_{\ell }{c}_{\ell }^{\dagger }{\tau }^{z}{c}_{\ell }+\frac{t}{2}\mathop{\sum}\limits_{\ell \ell ^{\prime} }^{\prime} {c}_{\ell }^{\dagger} {\tau }^{z}{c}_{\ell ^{\prime} }\\ 	+\frac{-i\lambda }{2}\mathop{\sum}\limits_{\ell \ell ^{\prime} }^{\prime} {c}_{\ell }^{\dagger} {\tau }^{x}{\hat{{\bf{n}}}}_{\ell \ell ^{\prime} }\cdot {\boldsymbol{\sigma }}{c}_{\ell ^{\prime} }+{m}_{\text{Z}}\mathop{\sum}\limits_{\ell }{(-)}^{{\ell }_{z}}{c}_{\ell }^{\dagger }{\sigma }^{z}{c}_{\ell }\ .$$

Here *ℓ* labels a lattice site **R**_*ℓ*_ = (*ℓ*_*x*_, *ℓ*_*y*_, *ℓ*_*z*_) on the unit cubic lattice, $\mathop{\sum }\nolimits_{\ell \ell ^{\prime} }^{\prime}$ indicates a sum over nearest neighbor sites, and ${\hat{{\bf{n}}}}_{\ell \ell ^{\prime} }^{\dagger }$ is the nearest neighbor unit vector. We have adopted an implied sum notation for the orbital and spin degrees of freedom, e.g., ${c}_{\ell }^{\dagger }{\tau }^{\mu }{\sigma }^{\nu }{c}_{\ell ^{\prime} }^{\dagger }={\sum }_{ij,st}{c}_{\ell is}^{\dagger }{\tau }_{ij}^{\mu }{\sigma }_{st}^{\nu }{c}_{\ell ^{\prime} \ jt}^{\dagger }$, where *τ* and *σ* are Pauli matrices for orbital and spin degrees of freedom respectively, and ${c}_{\ell is}^{\dagger }$ creates an electron on site *ℓ* in orbital *i* with spin *s*.

The first three terms in Eq. (8) correspond to the model of Bernevig et al.^63,64^ for a strong topological insulator, often written in *k*-space as ${H}_{\text{STI}}({\bf{k}})=m{\tau }^{z}+{\sum }_{i = x,y,z}t\cos ({k}_{i}){\tau }^{z}+\lambda \sin ({k}_{i}){\tau }^{x}{\sigma }^{i}$. In the last term in Eq. (8), *m*_Z_ is the strength of the staggered Zeeman field corresponding to A-type (layered) AFM order, doubling the unit cell and converting the model to represent an AFM topological insulator. Time reversal itself is now broken, but time reversal followed by a unit translation along $\hat{{\bf{z}}}$ is a good symmetry. For our choice of parameters, see Supplementary Methods, the model is in the topological phase, with a formal magnetoelectric coupling of (*θ*/2*π*)(*e*^2^/*h*) with axion coupling *θ* = *π*. As a result, $\hat{{\bf{z}}}$-normal surfaces are naturally gapped and carry an anomalous Hall conductivity of ±*e*^2^/2*h*.

### Wave-packet construction

We construct the initial WPs in the space of momentum *k*_∥_ along the direction of propagation. We calculate the surface band structure for a supercell Hamiltonian *H*_dw_ or *H*_st_ containing a domain wall or step, whose presence results in mid-gap bands localized on the conducting channels in the otherwise gapped surface, as shown in Fig. 1c,d, respectively. Note that technically each slab contains two domain walls and two steps. In the domain wall case the configuration as a whole is invariant under time reversal times inversion, so the bands shown are Kramers degenerate.

Next we construct the WP by making a quantum superposition of channel-localized solutions according to a *k*_∥_-space envelope function that we take to be a Gaussian. This results in a WP that is localized in all three real space dimensions. This is then used as the initial wave function Ψ(0) of the time-evolution problem for the much larger system that includes the QPJ and is described by the Hamiltonian *H*_QPJ_. The width of the WP is chosen narrow enough in momentum space so that it only samples the linear part of the dispersion in Fig. 1c, d. In this way we avoid fast spreading of the WP in real space. We have defined *H*_QPJ_ as the model Hamiltonian *H*_0_ in the presence of an antiferromagnetic domain wall and a single-height step that intersect in the center of the surface. A more detailed description of the WP construction can be found in the Supplementary Methods.

### Wave-packet dynamics

To avoid finite-size effects, we require the system size *L* to be much larger than the extent of the WPs along the channel. When both a domain wall and step are present, momentum is no longer a good quantum number in any direction, so we compute the time evolution entirely in real space. This is done using Chebyshev series expansion methods^65^ applied to the time-evolution operator *e*^−*iHt*^. We use slabs of size 160 × 160 in-plane and 16 cells thick, enough to minimize finite-size effects, and adopt a Chebyshev expansion order of *N*_C_ = 2^11^ so that we can time evolve the state accurately over the needed time intervals.

### STM tip modeling

To model the effects of the magnetic and electrostatic STM tips we extend the QPJ Hamiltonian (*H*_QPJ_) with two spatially dependent terms

9 $${\tilde{H}}_{\text{QPJ}}={H}_{\text{QPJ}}+{V}_{\text{Z}}\mathop{\sum}\limits_{\ell \in {{{\Omega }}}_{\text{Z}}}c_{\ell }^{\dagger }\,{\sigma }^{z}{c}_{\ell }+{V}_{\text{G}}\mathop{\sum}\limits_{\ell \in {{{\Omega }}}_{\text{G}}}c_{\ell }^{\dagger }\,{c}_{\ell }\ ,$$

where the second term modifies the Zeeman interaction in a region Ω_Z_ and the third term shifts the energy of all orbitals and spins uniformly inside a region Ω_G_.

For a positive *V*_Z_ in Eq. (9), we choose the region Ω_Z_ such that it restricts the sum to surface orbitals that lie within a radius *r* of the tip, and that already experience a negative Zeeman field from the bulk Hamiltonian of Eq. (8). Thus, *V*_Z_ = *m*_Z_ is just enough to remove the Zeeman field from these sites, and *V*_Z_ = 2*m*_Z_ makes the surface-layer Zeeman field equal on both sides of the domain wall or step, as in Fig. 4a. We can then tune between these extremes by taking *V*_Z_ ∈ [0, 2*m*_Z_], thus modeling cases in which the magnetic tip has only partially reversed the surface field. Similarly, for *V*_Z_ < 0, Ω_Z_ is chosen such that the second term in Eq. (9) is restricted to surface orbitals experiencing a positive Zeeman field in the bulk Hamiltonian.

The region of influence of the electrostatic tip, Ω_G_ in Eq. (9), is defined to be a rectangle centered at (*x*_0_, *y*_0_) relative to the QPJ and one unit cell deep, as shown by the gray shading in Fig. 4d. A WP propagating for a distance *ℓ* along any domain-wall or step channel lying inside the quantum well defined by Ω_G_ acquires an additional phase proportional to *ℓ*Δ*k*, where Δ*k* is the shift of the Fermi wavevector of the channel. In the approximation of linear dispersion, we have Δ*k* = *V*_G_/*ℏυ*_F_, where *V*_G_ corresponds to a local gate voltage and *υ*_F_ is the Fermi velocity (equal to *υ*_dw_ and *υ*_st_ for domain-wall and step channels respectively). Thus, the off-centering of Ω_G_ defined by (*x*_0_, *y*_0_) allows us to control the travel distances *ℓ* along each of the four “legs” near the junction, introducing extra phases that are given by

10 $$\alpha =-{{\Delta }}{k}_{\text{dw}}{y}_{0},\,\gamma =-{{\Delta }}{k}_{\text{st}}{x}_{0}$$

in Eq. (7).

## Supplementary information

Supplementary Information

Description of Additional Supplementary Files

Supplementary Movie 1

Supplementary Movie 2

Supplementary Movie 3

Supplementary Movie 4

Supplementary Movie 5

Supplementary Movie 6

## Data Availability

The data sets generated and/or analyzed during the current study are available from the corresponding author on reasonable request.

## Code availability

The model system and wave packets were constructed the open-source code package PythTB http://www.physics.rutgers.edu/pythtb/. The code used to perform wave-packet dynamics is available upon request.
