# Supplementary material for: Controllable quantum point junction on the surface of an antiferromagnetic topological insulator
Source: Nat Commun. 2021 Jun 28;12:3998. doi: 10.1038/s41467-021-24276-5 (PMC8238970; doi:10.1038/s41467-021-24276-5)
Supplement: Supplementary file 1 — Supplementary Information [file 41467_2021_24276_MOESM1_ESM.pdf]

Controllable quantum point junction on the surface of an antiferromagnetic topological insulator
Supplementary Information

Nicodemos Varnava,[1,*] Justin H. Wilson,[1] J. H. Pixley,[1,2,3] and David Vanderbilt[1]

[1]*Department of Physics & Astronomy, Center for Materials Theory, Rutgers University, Piscataway, New Jersey 08854, USA*
[2]*Center for Computational Quantum Physics, Flatiron Institute, 162 5th Avenue, New York, NY 10010*
[3]*Physics Department, Princeton University, Princeton, New Jersey 08544, USA*

I. SUPPLEMENTARY METHODS

A. Bulk and surface states

For completeness we repeat the bulk Hamiltonian

$$H_0 = m\sum_{\ell} c_\ell^\dagger \tau^z c_\ell + +m_{\rm Z}\sum_{\ell}(-)^{\ell_z} c_\ell^\dagger \sigma^z c_\ell + \frac{t}{2}{\sum_{\ell\ell'}}' c_\ell^\dagger \tau^z c_{\ell'} + \frac{-i\lambda}{2}{\sum_{\ell\ell'}}' c_\ell^\dagger \tau^x \hat{\mathbf{n}}_{\ell\ell'}\cdot\boldsymbol{\sigma} c_{\ell'} \,. \tag{1}$$

From left to right, the four terms describe the onsite energies, staggered Zeeman field, and spin-independent and spin-depended nearest-neighbor hoppings. We first consider the case where $m_Z = 0$, in which case the Hamiltonian H_0 reduces to the time-reversal ($\mathcal{T}$) symmetric model proposed by Bernevig et al.[1,2] $\mathcal{T}$ symmetry is an axion-odd symmetry, meaning the axion coupling is quantized to $\theta = 0$ or π in its presence. At half-filling and for $(m, t, \lambda) = (1.0, -0.5, -0.6)$, the ground state has $\theta = \pi$, corresponding to a strong topological insulator (STI). The bulk-boundary correspondence then implies the presence of $\mathcal{T}$-protected surface Dirac cones, as illustrated in Supplementary Fig. **1c**, for the (001) surface.

For $m_Z = -0.3$, even though $\mathcal{T}$ is broken, $\mathcal{T}$ followed by a half-lattice translation along $\hat{\mathbf{z}}$ ($\mathcal{T} * \tau_{1/2}$) is a good symmetry. This symmetry is axion-odd as well[3,4] and $\theta = \pi$ for the above choice of parameters, making it an AFM-TI insulator. An important difference between $\mathcal{T}$ and $\mathcal{T} * \tau_{1/2}$ is that the latter symmetry does not force the surface AHC of all surfaces to vanish. In fact, Supplementary Fig. **1f**, shows that the AFM Zeeman term opens the (001) surface gap, with the top and bottom surfaces exhibiting a half-integer $(n + 1/2)e^2/h$ AHC, where n depends on the surface termination[5].

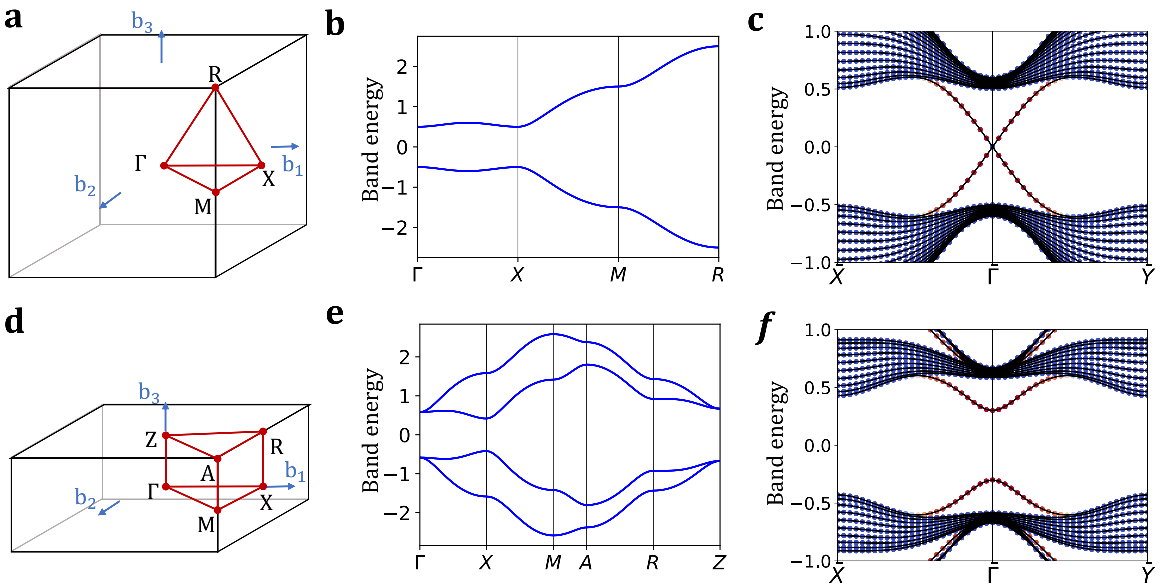

Supplementary Figure 1. **Bulk and surface states.** **a**, Brillouin zone, **b**, bulk bands and **c**, surface bands along (001) for the STI model. **d-f** Same for the AFM-TI model. In **b**, **e**, the bands are doubly degenerated due to time-reversal composed with inversion $\mathcal{T} * \mathcal{I}$ and in **c**, **f**, red/blue markers indicate surface/bulk bands.

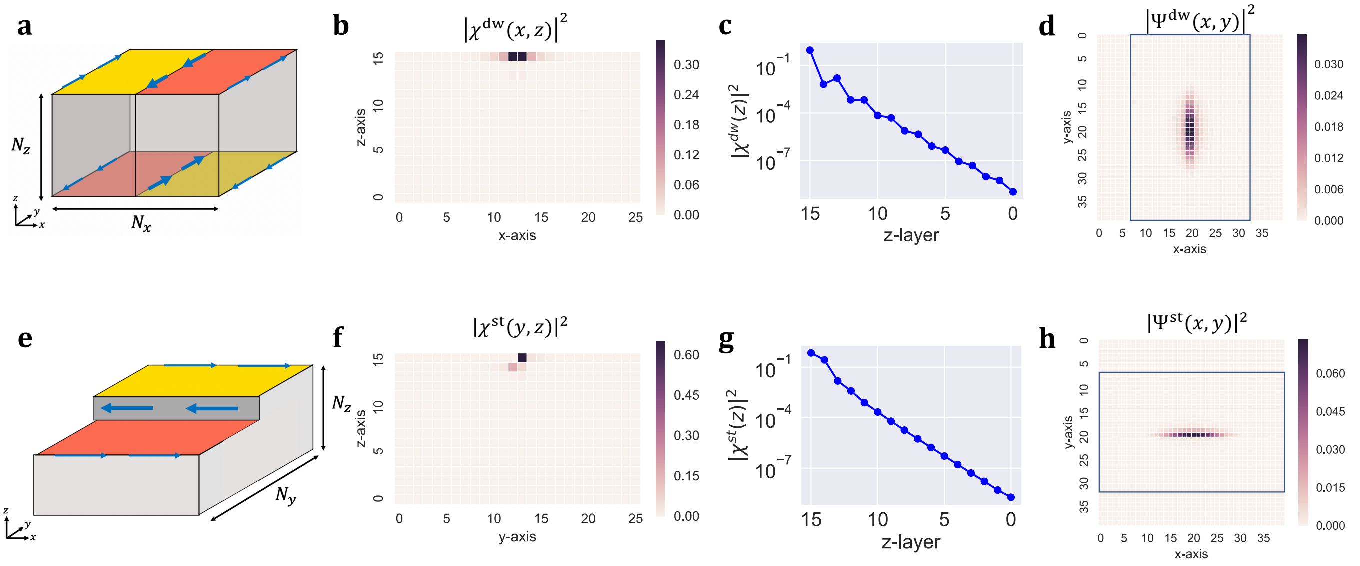

Supplementary Figure 2. **Wave packet construction.** **a**, Sketch of the AFM-TI supercell slab with a domain-wall in direction y and the associated energy states. **b**, Spatial profile of the transverse shape of the WP $|\chi^{\rm dw}(x,z)|^2 = \sum_{\sigma\tau} |\chi^{\rm dw}_{\sigma\tau}(x,z)|^2$. **c**, Logarithmic plot of the layer density $|\chi^{\rm dw}(z)|^2 = \sum_x |\chi^{\rm dw}(x,z)|^2$ which shows the exponential localization of the states at the surface. **d**, The initial (001)-projected domain-wall WP $|\Psi^{\rm dw}(x,y)|^2 = \sum_{\sigma\tau,z} |\Psi^{\rm dw}_{\sigma\tau}(x,y,z)|^2$. Note that the constructed WP (inside the box) has been placed in a larger system with zero amplitude assigned outside the box. **e-h**, Same as **a-d**, but for a step along the x direction. Comparing **b** and **f**, we see that in the case of a step the surface state wavefunction redistributes itself across the two surfaces in an unequal manner.

B. Wave packet construction

For clarity we focus on the construction of domain-wall channel WPs, since the construction of step channel WPs follows in a similar fashion. We start by considering an $N_x \times 1 \times 1$ supercell of the AFM-TI model and use it to create a slab that is N_z unit cells thick along (001). Because of the periodic boundary conditions, we have no choice but to create two domain walls. We choose these to be centered at the $x = 1/2$ and $(N_x + 1)/2$ planes, and create them by flipping the Zeeman potential of all orbitals in the half-cell to the left of the $x = 1/2$ plane. We refer to the Hamiltonian of this slab supercell with a pair of domain walls as $\hat{H}_{\rm dw}$. Supplementary Fig. **2a**, shows a sketch of the slab supercell while the associated energy bands were presented in the main text Fig. **1c** for a supercell with $N_x = 20$ and $N_z = 8$. We see that two counter-propagating, doubly degenerate, linear bands appear in the insulating gap, corresponding to the four chiral channels indicated with blue arrows in Supplementary Fig. **2a** (note the in-plane periodic boundary conditions).

The Bloch eigenvectors of $\hat{H}_{\rm dw}$ are $\psi^{\rm dw}_{nk_y\sigma\tau}(\mathbf{r}) = e^{ik_y y}\, u^{\rm dw}_{nk_y\sigma\tau}(x,z)$, where $u^{\rm dw}_{nk_y\sigma\tau}$ are the cell-periodic counterparts. Note that we neglect the exponentially small dispersion along the k_x-direction, and the y dependence is absent from $u^{\rm dw}_{nk_y\sigma\tau}$ because there is only a single site per unit cell. A technical difficulty arises from the fact that the chiral channels are doubly degenerate as a result of the $I * T$ symmetry of $\hat{H}_{\rm dw}$, but we extract channel-localized states by diagonalizing the z operator in the space of the two degenerate states. We denote the channel-localized states as $\tilde{u}^{\rm dw}_{\nu k_y\sigma\tau}(x,z)$, where the index ν labels the four chiral channels.

The final step in constructing our initial WPs is to take quantum superpositions of channel-localized Bloch states $\tilde{\psi}^{\rm dw}_{\nu k_y\sigma\tau}(\mathbf{r})$ according to a Gaussian envelope function $F(k_y) = A\exp\left(-k_y^2/2\kappa^2\right)$ centered at $k_y = 0$, where A is a normalization factor and κ is a measure of the extent of the WP along the channel. Our initial WPs for the domain-wall are then

$$\Psi^{\rm dw}_{\sigma\tau}(\mathbf{r}, t=0) \;=\; \int_{-\pi}^{\pi} dk_y\, F(k_y)\, \tilde{\psi}^{\rm dw}_{\nu k_y\sigma\tau}(\mathbf{r}). \tag{2}$$

At this point we make the additional approximation $\tilde{u}^{\rm dw}_{\nu k_y\sigma\tau}(x,z) = \tilde{u}^{\rm dw}_{\nu 0\sigma\tau}(x,z)$, which is well justified for a WP of sufficiently narrow extent in k_y. We note that this approximation is equivalent to the WP decomposition of Eq. (1)

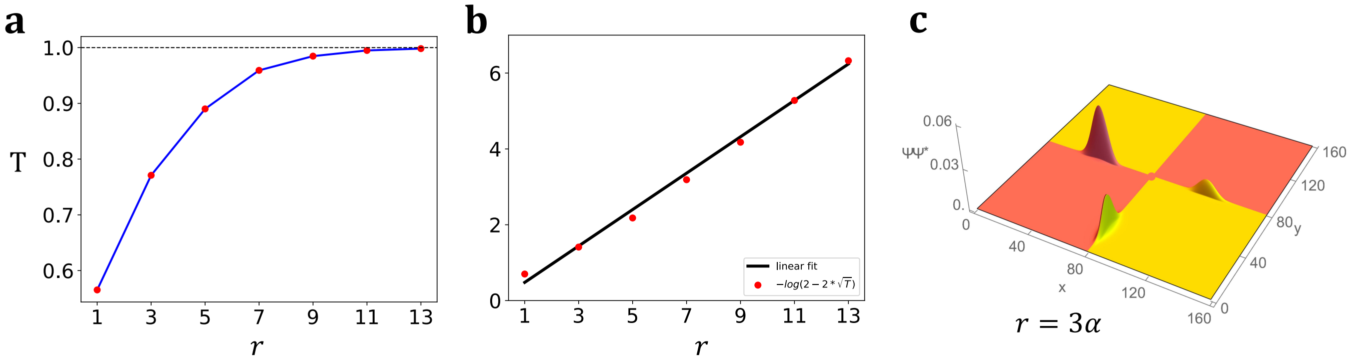

Supplementary Figure 3. **Tunneling between decoupled channels.** **a**, Transmission as a function of the radius of the polarized region. The radius is measure in units of the lattice constant α. **b**, Linearizing the plot in **a**, to extract the tunneling length ξ. **c**, Example of tunneling for polarized region with $r = 3\alpha$.

in the main text with the identification

$$\chi^{\mathrm{dw}}_{\sigma\tau}(x,z) = \tilde{u}^{\mathrm{dw}}_{\nu 0\sigma\tau}(x,z), \qquad f(r) = \int_{-\pi}^{\pi} dk\, e^{ikr} F(k). \tag{3}$$

The WP construction for the case of the step channel follows in a similar way. Because the steps only need to be created at the top surface of the slab, the channel-localized states are nondegenerate. We note that the periodic boundary conditions enforce a second step channel, which produces states of both chirality in the dispersion shown in Fig. 1**d**, of the main text. The rest of the above discussion applies, but with $x \leftrightarrow y$ and $k_x \leftrightarrow k_y$ because we take the steps to propagate along $\hat{x}$.

II. SUPPLEMENTARY DISCUSSION

A. Channel tunneling

In the main text we demonstrated the control of magnitude splitting by applying the magnetic tip in a circular region (of constant radius) centered at the junction and varying the strength V_Z of the tip (see Eq. 9). Here instead we apply a magnetic tip with constant $V_{\mathrm{Z}} = 2m_{\mathrm{Z}}$, i.e., strong enough to polarize the spins and decouple the channels, and we vary the radius of the region applied. In this way, we are effectively modeling a quantum point contact since a WP can scatter to the decoupled channel only through tunneling. Furthermore, this approach enables the extraction of the *tunneling length* ξ.

In Supplementary Fig. 3**a**, we plot the transmission $T = \cos^2\beta/2$, i.e., the magnitude of the wave function that has remained on the channel, to show the exponential suppression of tunneling to the decoupled channel. Since, $T(r=0) = 0.5, T(r \to \infty) = 1.0$ we heuristically expect $T \simeq 1 - e^{-2r/\xi}/2$ with the 2 in exponent coming from the fact that T is related to the square of the wavefunction. In Supplementary Fig. 3**b**, we replot the data on rescaled axes to extract the tunneling length, which is approximately $\xi = 0.48\alpha$.

B. Breaking symmetries

The simple model Hamiltonian we are using has many symmetries. These extra symmetries do not affect any of the core features of our proposal such as the unidirectionality and topological protection of the channels or the robustness and controllability of the junction. However, they result in some features that should not be generically expected in any realistic implementation. In this section we add symmetry breaking terms to the Hamiltonian and illustrate how these non-essential features are affected. In particular, we find that mirror-symmetry-breaking terms affect the amplitude splitting and particle-hole-symmetry-breaking terms the group velocities of the domain-wall and step channels.

a. Mirror symmetry As discussed in the main text, the fact that the QPJ "naturally" implements the Hadamard gate is a manifestation of the high symmetry of the model and of the geometry of the junction. To this effect we

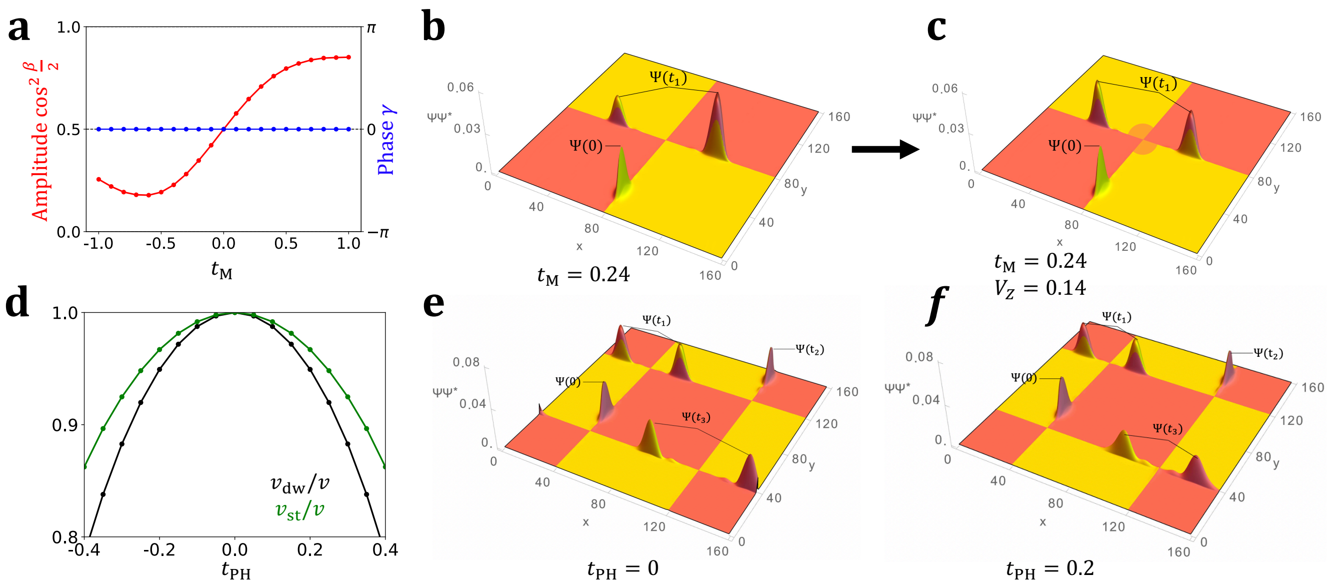

Supplementary Figure 4. **Effect of symmetry breaking.** **a** − **c**, A mirror-symmetry-breaking term modifies the magnitude splitting at the junction **d** − **f**, A particle-hole-symmetry-breaking term enhances the group-velocity anisotropy of the domain-wall and step channels. **a**, Magnitude $\cos^2(\beta/2)$ and phase γ (defined in Eq. (7) of the main text) as a function of the strength t_M of the mirror-breaking hopping term V_M . Only the magnitude is modified by the addition of this term. **b**, Setting $v = 0.24$ in Supplementary Equation (4) causes unequal splitting of the WP but we can use the magnetic tip with $V_Z = 0.14$ **c**, to cancel the effect of the mirror-breaking term. **d**, Group velocity of the domain-wall and step channel as a function of the strength t_{PH} of mirror-breaking term V_{PH}. Time-evolution without **e**, and with **f**, the inclusion of V_{PH}. The effect is to slow down the WP but has no effect on the S-matrix.

break the extraneous M_x and M_y mirror symmetries and study how they affect the S-matrix. To do that we add the term

$$V_M = \frac{-it_M}{2} \sum_{\ell\ell'}{}' c_\ell^\dagger \tau^x \sigma^z c_{\ell'} \delta_{\ell_z \ell'_z} \ , \tag{4}$$

where $\delta_{\ell_z \ell'_z}$ ensures that only in-plane hopping terms are considered. Note that this term couples spin and orbit in a way that preserves $\mathcal{T}$ symmetry[6]. Even though Supplementary Equation (4) does not affect the magnetic properties of the junction it nonetheless affects the S-matrix by modifying the magnitude splitting $\cos^2(\beta/2)$, as the numerical calculations indicate in Supplementary Fig. **4a**. Importantly, the universal control over the S-matrix magnitudes using the local magnetic tip enables us to eliminate (i.e., calibrate) the effect of the mirror-symmetry-breaking terms, as illustrated in Supplementary Fig. **4b**.

b. Particle-hole symmetry A secondary feature of the model is that the group velocities of the domain-wall and step channels are approximately equal $v_{dw} \approx v_{st} = v$ (less than 1% difference). We can enhance the anisotropy by including a particle-hole-symmetry-breaking term

$$V_{PH} = \frac{t_{PH}}{2} \sum_{\ell\ell'}{}' c_\ell^\dagger c_{\ell'} \delta_{\ell_x \ell'_x} \delta_{\ell_y \ell'_y} \ , \tag{5}$$

where again we make use of delta functions to ensure only hoppings along specific directions are considered, in this case only along the z-direction. Supplementary Fig. **4d**, shows that the effect of V_{PH} is to reduce the group velocities of both channels in an inequivalent way.

It is important to realize that for any junction, both of the incoming (or outgoing) channels are of the same type, either both domain-wall or both step. Thus, even if the velocities of the two channels are unequal, this will not cause a relative delay between the arrival of the two wave packets at the junction which would affect their interference. This is also confirmed by the explicit time-evolution simulations in Supplementary Fig. **4e**, **f**, where we illustrate how the addition of V_{PH} slows down the WPs but does not effect any of our conclusions.

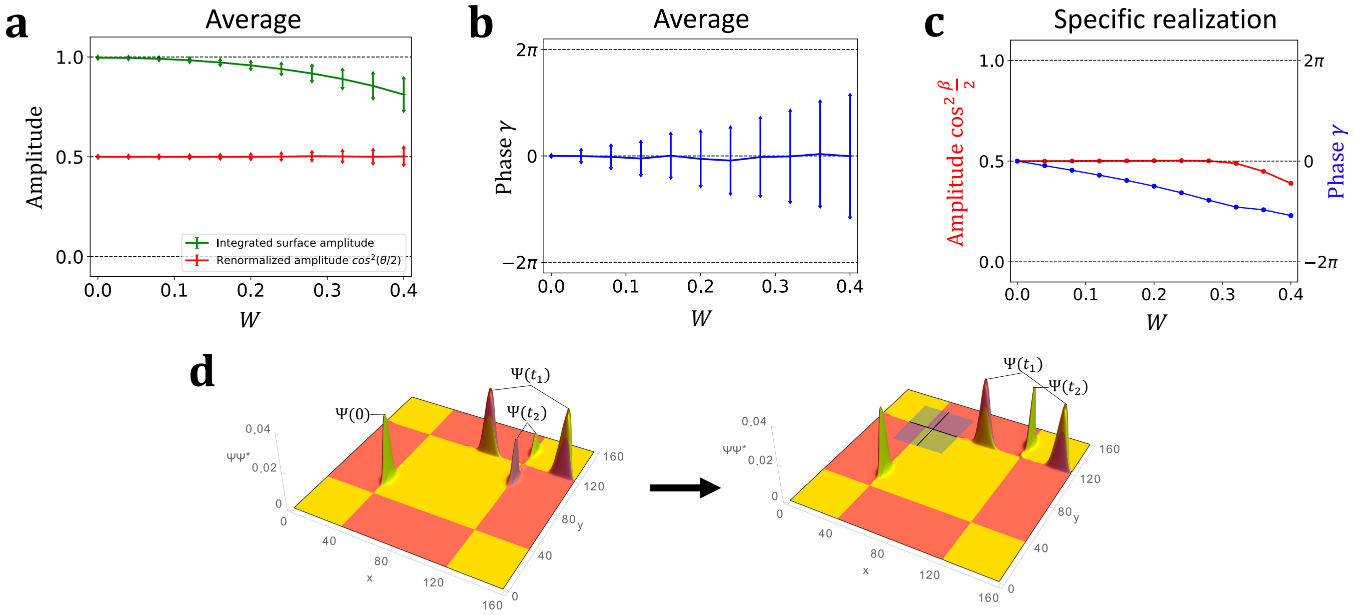

Supplementary Figure 5. **Effect of disorder.** **a**, **b**, Averaged magnitude splitting $\cos^2(\theta/2)$ and phase γ over 20 disordered configurations for different values of W. **a**, Because the initial WP $\Psi(0)$ is not an exact eigenstate there is loss to the bulk we need to take into account. The green line shows the magnitude of the WP that remained on the surface after scattering at Junction 1 and the red line corresponds to the renormalized magnitude splitting. The disorder affects slightly the magnitude splitting which fluctuates about 50% for different realizations of disorder. **b**, The phase γ is significantly affected by disorder and needs to be accounted. **c**, Example of a specific disorder realization as a function of W. **d**, For the realization in **c**, with $W = 0.1$ we can use the gate voltage tip to eliminate the random phase difference accumulated up to $t = t_2$, due to the disorder. In this example we set the strength of the gate voltage to $V_{\mathrm{G}} = 0.1$ and the center of the rectangle at $(x_0, y_0) = (8, 0)$.

C. Stability to disorder

Finally we show that the QPJ is robust in the presence of disorder. We introduce disorder into the model Hamiltonian by adding a random potential scattering term given by

$$V_{\mathrm{D}} = \sum_{\ell} m_{\mathrm{D}}(\ell) c_\ell^\dagger c_\ell \; . \tag{6}$$

The disorder potential $m_D(\ell)$ is sampled from a Gaussian distribution at each site in the three-dimensional lattice ℓ with zero mean and standard deviation W, which characterizes the strength of the disorder potential. For sufficiently large W the average band gap in the bulk will close and the model will transition out of a topological phase. In contrast, for weaker disorder strengths (relative to the clean band gap, which is equal to $E_g \approx 0.6$ here) the topological properties are expected to remain robust. This should also provide a level of protection of the chiral surface states from back-scattering due to the disorder potential, but how this impacts the quantum point junction remains unclear.

To demonstrate the robustness of the junction to disorder, we have performed a similar analysis as in the previous section. In contrast with Supplementary Discussion Sec. II B, we cannot construct the initial WP from the exact eigenstates because of the presence of disorder in the system. For that reason, we use the same initial WP that was constructed for the clean model, and renormalize our results by accounting for the prompt loss of amplitude associated with the fact that this trial function has nonzero overlap with some extended bulk and surface states (see Supplementary Fig. **5a**). The results are averaged over 20 disorder samples and show that magnitude and phase evolution through the junction are randomly effected by disorder, and therefore retain the same average value as they do in the absence of disorder. In particular, the random potential induces both a splitting of the WP magnitude as well as a shift in the phase that are both random for each sample, which clearly averages to zero as demonstrated in Supplementary Fig. **5a**, **b**.

Lastly, we turn to how disorder can impact the QPJ in a specific disorder realization. We show that the effect of disorder can be calibrated adopting an approach like that used above for the mirror-breaking term. As shown in Supplementary Fig. **5c**, for a specific random sample, the disorder does not affect the splitting, at least for small W, while the phase of the WP is significantly different from the clean limit. We then apply the gate voltage to completely

remove this effect, returning the QPJ to its clean behavior, Supplementary Fig. **5d**. These results demonstrate both a robustness and a level of control over disorder in this novel QPJ. This is important, as each device made out of such a QPJ will have some random disorder profile. Nevertheless, as we have shown in Supplementary Fig. **5**, the electrostatic STM tip can be used to remove this effect, returning the QPJ to its ideal behavior.

---

* nvarnava@physics.rutgers.edu

[1] B. A. Bernevig, T. L. Hughes, and S.-C. Zhang, Science **314**, 1757 (2006).
[2] T. L. Hughes, E. Prodan, and B. A. Bernevig, Phys. Rev. B **83**, 245132 (2011).
[3] R. S. K. Mong, A. M. Essin, and J. E. Moore, Phys. Rev. B **81**, 245209 (2010).
[4] N. Varnava, I. Souza, and D. Vanderbilt, Phys. Rev. B **101**, 155130 (2020).
[5] N. Varnava and D. Vanderbilt, Phys. Rev. B **98**, 245117 (2018).
[6] B. J. Wieder and B. A. Bernevig, The axion insulator as a pump of fragile topology (2018), arXiv:1810.02373.
