# Supplementary material for: Controllable quantum point junction on the surface of an antiferromagnetic topological insulator
Source: Nat Commun. 2021 Jun 28;12:3998. doi: 10.1038/s41467-021-24276-5 (PMC8238970; doi:10.1038/s41467-021-24276-5)
Supplement: Supplementary file 2 — Description of Additional Supplementary Files [file 41467_2021_24276_MOESM2_ESM.docx]

File Name: Supplementary Movie 1
Description: Wave packet evolution animation corresponding to figure 3a in the main text.

File Name: Supplementary Movie 2
Description: Wave packet evolution animation corresponding to figure 3e in the main text.

File Name: Supplementary Movie 3
Description: Wave packet evolution animation corresponding to figure 4a in the main text.

File Name: Supplementary Movie 4
Description: Wave packet evolution animation corresponding to figure 4b in the main text.

File Name: Supplementary Movie 5
Description: Wave packet evolution animation corresponding to figure 4d in the main text.

File Name: Supplementary Movie 6
Description: Wave packet evolution animation corresponding to figure 4e in the main text.
