# Supplementary figures and images for: Controllable quantum point junction on the surface of an antiferromagnetic topological insulator
Source: Nat Commun. 2021 Jun 28;12:3998. doi: 10.1038/s41467-021-24276-5 (PMC8238970; doi:10.1038/s41467-021-24276-5)

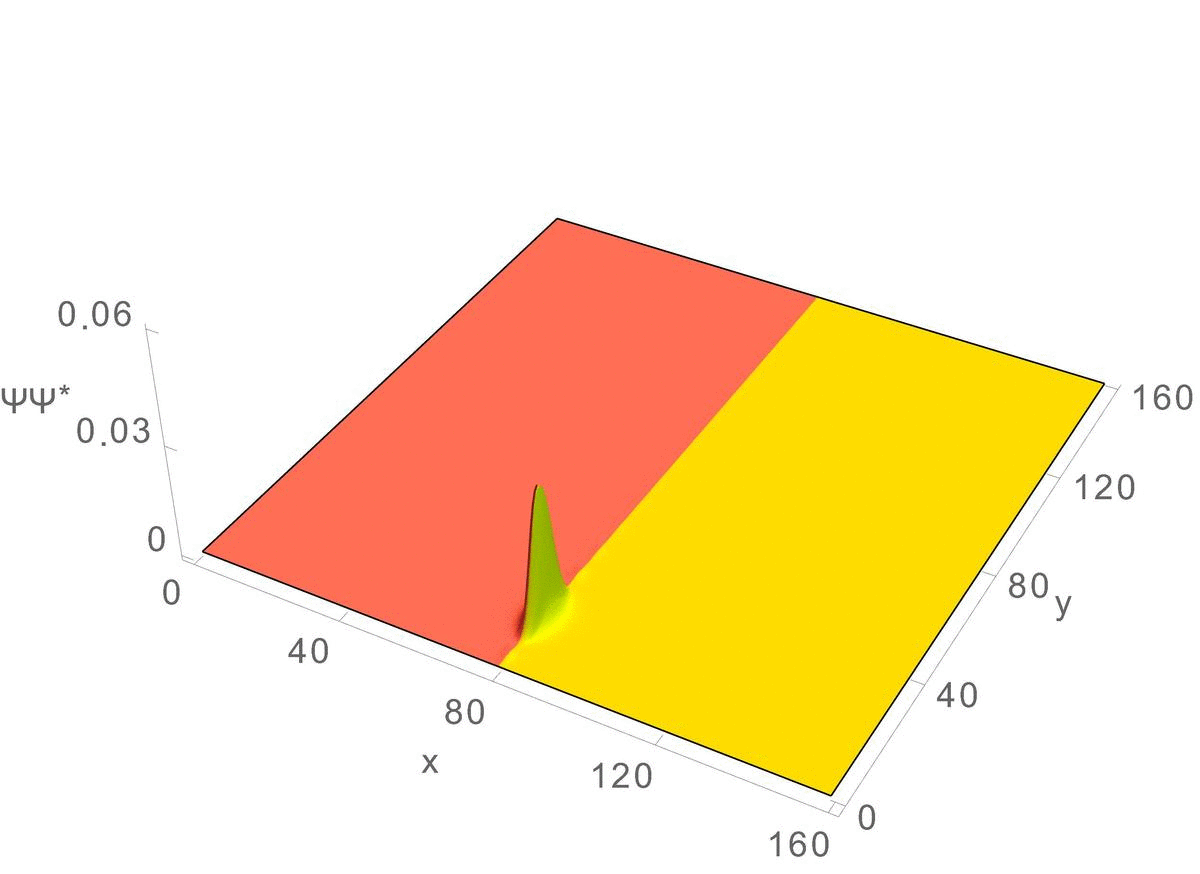

Supplement: Supplementary file 3 — Supplementary Movie 1 [file 41467_2021_24276_MOESM3_ESM.gif]

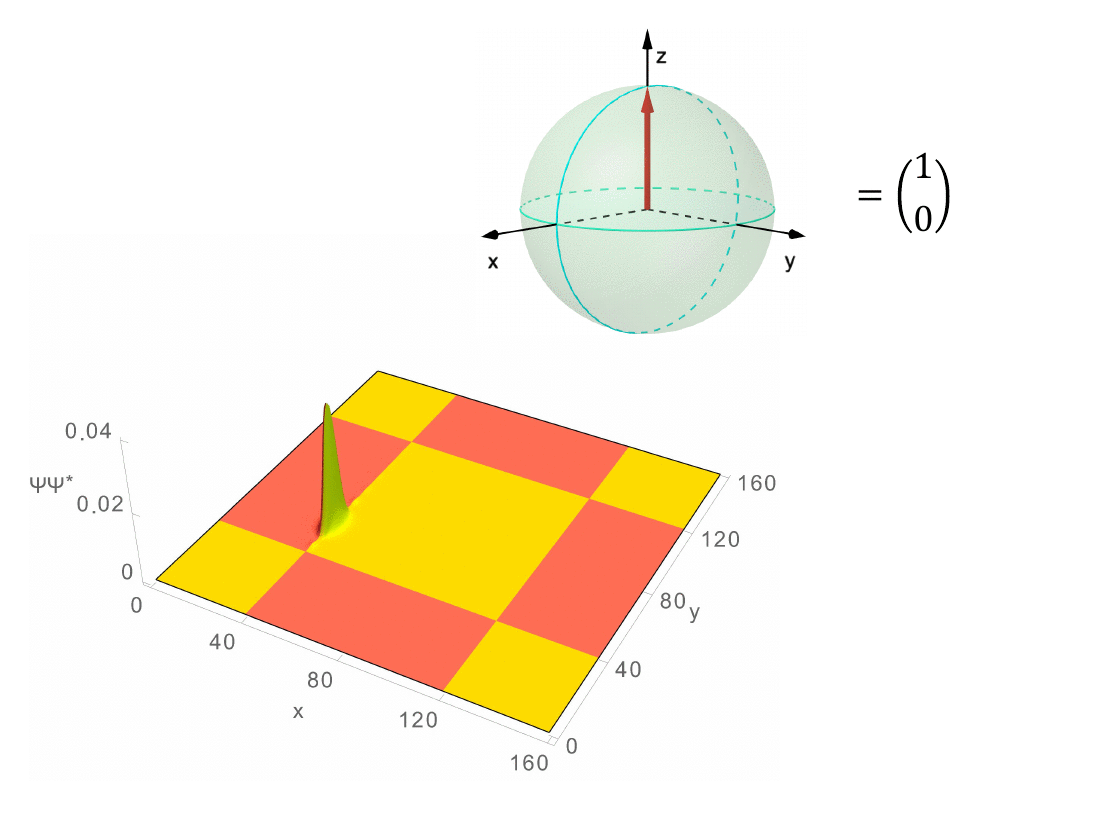

Supplement: Supplementary file 4 — Supplementary Movie 2 [file 41467_2021_24276_MOESM4_ESM.gif]

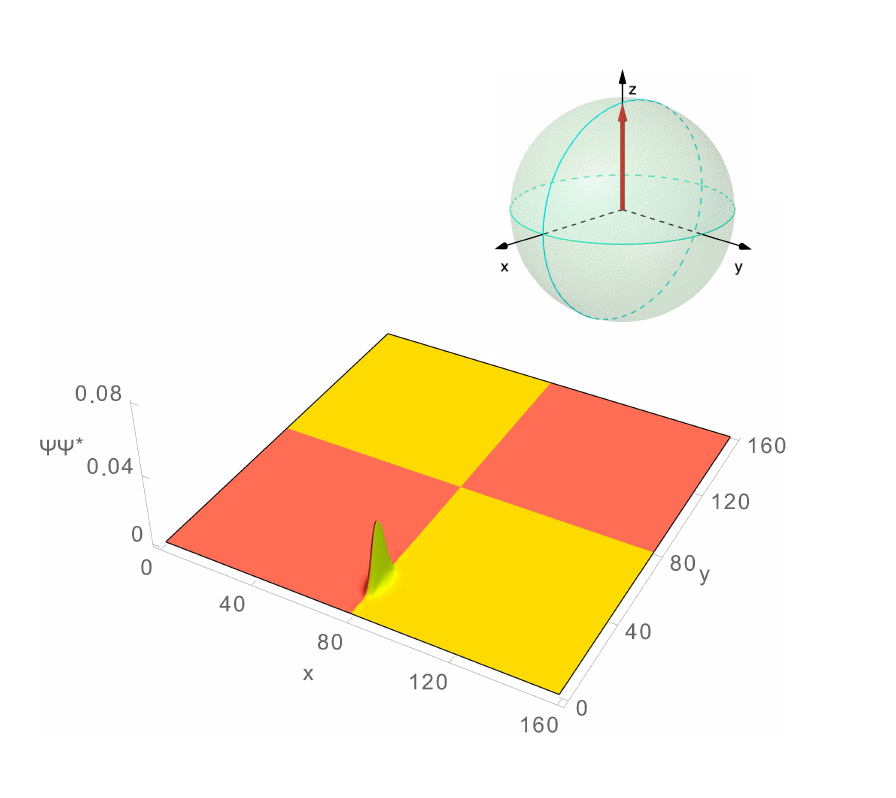

Supplement: Supplementary file 5 — Supplementary Movie 3 [file 41467_2021_24276_MOESM5_ESM.gif]

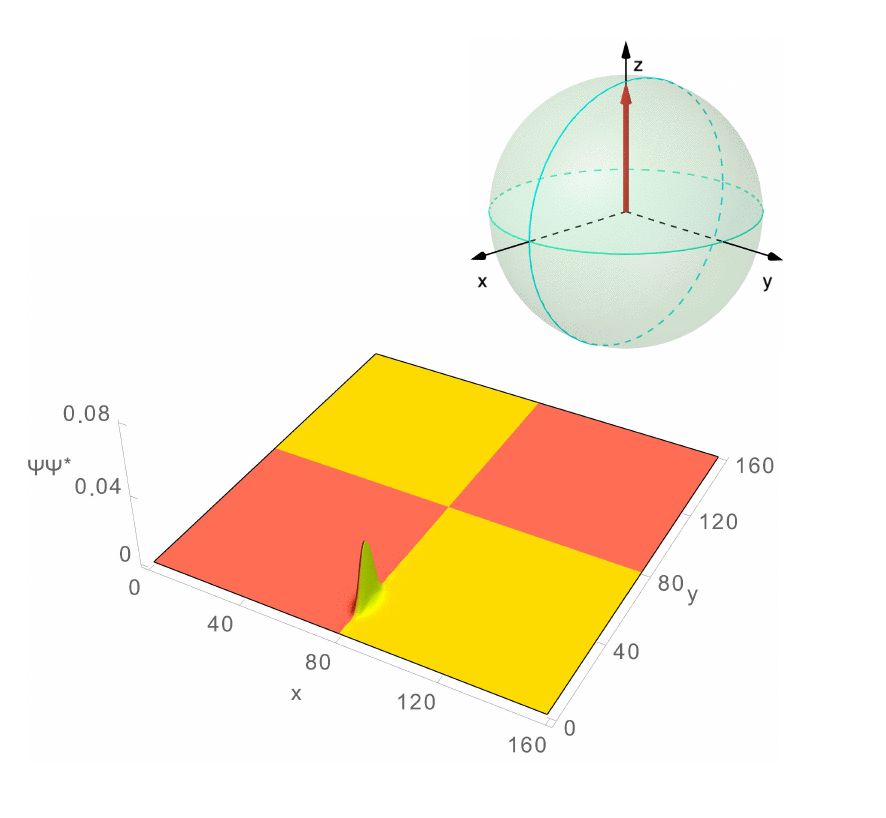

Supplement: Supplementary file 6 — Supplementary Movie 4 [file 41467_2021_24276_MOESM6_ESM.gif]

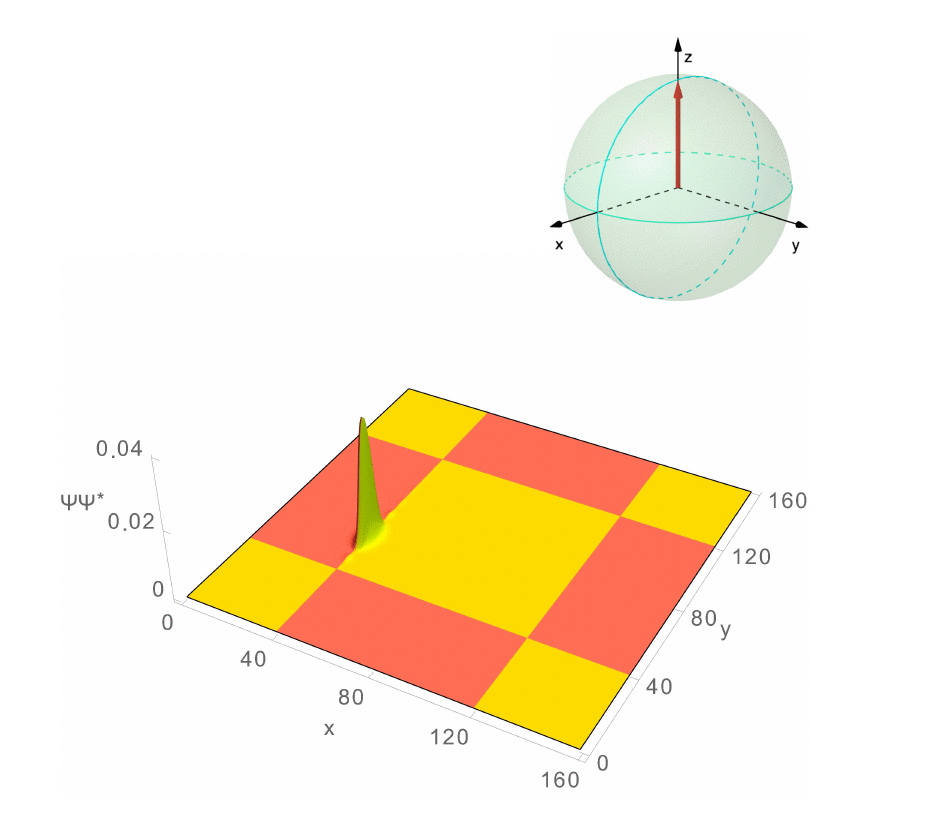

Supplement: Supplementary file 7 — Supplementary Movie 5 [file 41467_2021_24276_MOESM7_ESM.gif]

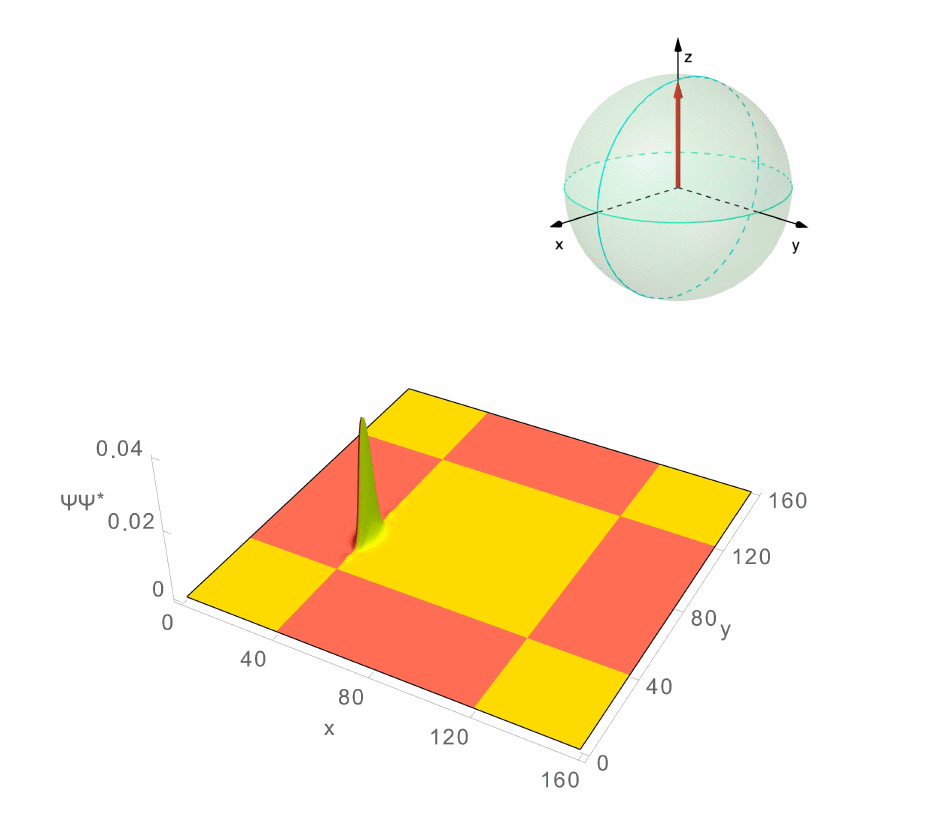

Supplement: Supplementary file 8 — Supplementary Movie 6 [file 41467_2021_24276_MOESM8_ESM.gif]
